# The complete mitochondrial genome of the dusky brown-gray–colored honeybee, *Apis mellifera* (insecta: Hymenoptera: Apidae) of New Zealand

**DOI:** 10.1080/23802359.2018.1507641

**Published:** 2018-10-29

**Authors:** Mito Maeda, Ikumi Nakagawa, Mao Chikano, Hisashi Okuyama, Robert Murray, Jun-Ichi Takahashi

**Affiliations:** a Department of Life sciences, Kyoto Sangyo University, Kyoto, Japan;; b Tai Tokerau Honey Ltd, Kaitaia, New Zealand

**Keywords:** Dusky brown-gray honeybee, carniolan, *Apis mellifera carnica*, New Zealand, subspecies

## Abstract

We analyzed the complete mitochondrial genome of the dusky brown-gray–colored honeybee *Apis mellifera*, collected from North Island, New Zealand. We determined that the mitochondrial genome was a 16,336 bp and predicted 13 protein-coding genes (PCGs), 22 tRNA genes, and two rRNA genes. The start codon ATA was found in two genes, ATG in four genes, ATT in six genes, and ATC in one gene, whereas the termination codon TAA was observed in all PCGs. The non-coding regions of *tRNA-Leu* and *COII* were consistent with the C haplotype of *A. mellifera carnica*. Phylogenetic analysis suggests a close relationship with the European *A. mellifera*.

The western honeybee, *Apis mellifera,* is naturally distributed in Africa, the Middle East, and Europe, and is exported worldwide. In New Zealand, the European honeybee subspecies *A. m. mellifera* was first introduced from the UK in 1839 (Beard 2015). Later, two subspecies, *A. m. carnica* and *A. m. ligustica*, were introduced to New Zealand from Europe and Australia. Import of honeybees is now prohibited to control the spread of disease (Beard 2015). Currently, there are three types of honeybees in New Zealand, each with different body colors. Yellow-colored honeybees (*A. m. ligustica*) have an Italian origin, as shown by the analysis of complete mitochondrial DNA (Nakagawa et al. 2018). Here, we report the complete mitochondrial genome sequence of the New Zealand dusky brown-gray–colored honeybee, *A. mellifera*. This taxon provides an important sample for rigorous phylogenetic studies of New Zealand honeybees and other *A. mellifera* subspecies.

Adult workers were collected in March 2017 from Kaitaiya, North Island, New Zealand (−35^°^11′45, 173^°^25′39). Genomic DNA was isolated from one worker and sequenced using Illumina’s HiSeq platform (Illumina Inc., San Diego, CA). We generated a DNA library of individuals from New Zealand that consisted of 1,879,764 reads. The resultant reads were assembled and annotated using the MITOS web server (Germany; Bernt et al. 2013) and Geneious R9 (Biomatters, New Zealand). A phylogenetic tree was constructed using MEGA6 (Tamura et al. 2013) and TREEFINDER (Jobb 2015) using the nucleotide sequences of 13 protein-coding genes (PCGs). The DNA specimen was stored in the National Museum of Nature and Science, Japan, accession number: NSMT-I- HYM 75324.

The dusky brown-gray–colored *A. mellifera* mitochondrial genome was determined to be 16,336 bp long (AP018434). This size is typical for a hymenopteran species. It was found to be similar to the common *A. mellifera* mitochondrial genome organization, comprising 13 PCGs, 22 putative tRNA genes, two rRNA genes, and an A + T-rich control region. The average AT content of the *A. mellifera* mitochondrial genome was 84.95%. Similar to the honeybee mitochondrial genomes, the heavy strand encoded nine protein-coding genes and 14 tRNA genes, and the light strand encoded four protein-coding genes, eight tRNAs, and two rRNA genes. *ATP6* and *ATP8* shared 19 nucleotides in common. Six protein-coding genes of the *A. mellifera* mitochondrial genome started with *ATT*, while *ATP6*, *COIII, ND4* and *Cytb* started with ATG, and *COI* and *ND3* started with ATA. *ND2* stared with ATC. This pattern is commonly found in *A. mellifera* subspecies (Crozier and Crozier 1993; Gibson and Hunt 2015; Haddad 2015; Hu et al. 2015; Eimanifar et al. 2016a, 2016b, 2017a, 2017b, 2017c; Haddad et al. 2017; Nakagawa et al. 2018). The stop codon of these genes was uniformly TAA, similar to other honeybee subspecies. All the tRNA genes possessed inferred cloverleaf secondary structures, except for *Gln*, *Ser1*, and *Thr*, which lacked the arm structure. Phylogenetic analysis was conducted using 13 mitochondrial PCG sequences from 24 closely related taxa (Figure 1). The New Zealand dusky brown-gray–colored *A. mellifera* was found to be most closely related to the European honeybee.

**Figure 1.**
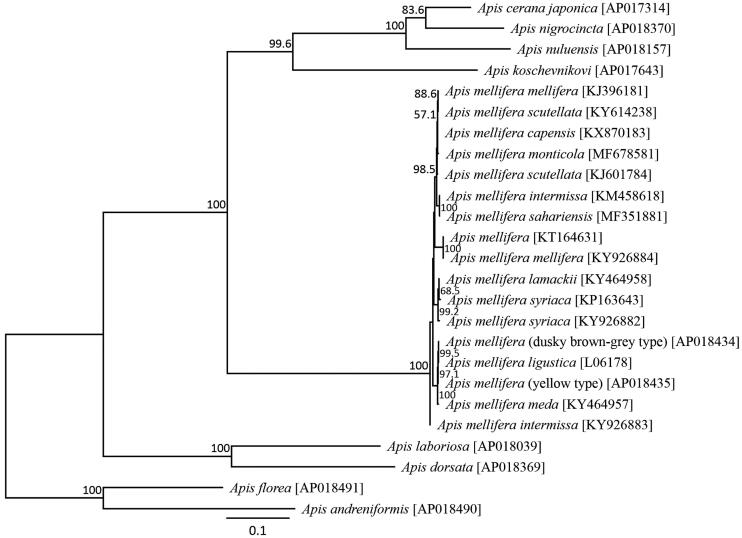
Inferred phylogenetic relationships of the genus *Apis* (Hymenoptera) using mitochondrial sequences from 13 protein-coding genes under the maximum likelihood criterion. Numbers at the nodes indicate bootstrap support (1,000 replicates). *A. florea, A. andreniformis, A. dorsata, A. laboriosa, A. cerana, A. nigrocincta,* and *A. koschevnikovi* (Takahashi et al. 2016, 2017a, 2017b, 2017c, 2017d, 2018; Wakamiya et al. 2017) were used as an outgroup. The alphanumeric terms in the parentheses indicate the GenBank accession numbers.
